# Estimation of global rating changes in quality of life and marital satisfaction among reproductive age women in Iran and Afghanistan before and after COVID-19 pandemic

**DOI:** 10.1186/s12905-023-02467-0

**Published:** 2023-06-16

**Authors:** Masomeh Khalili, Shahideh Jahanian Sadatmahalleh, Malihe Nasiri, Ali Montazeri

**Affiliations:** 1 Department of Midwifery and Reproductive Health, Faculty of Medical Sciences, Tarbiat Modares University, Tehran, Iran; 2 Department of Basic Sciences, School of Nursing and Midwifery, Shahid Beheshti University of Medical Sciences, Tehran, Iran; 3 Health Metrics Research Center, Iranian Institute for Health Sciences Research, ACECR, Tehran, Iran; 4 Faculty of Humanity Sciences, University of Science and Culture, Tehran, Iran

**Keywords:** Quality of life, Marital satisfaction, COVID-19, Women of reproductive age

## Abstract

**Background:**

Quality of life and marital satisfaction are important components of reproductive-age women’s health. This study aimed to compare the quality of life and marital satisfaction in women of reproductive age in Iran and Afghanistan before and after the COVID-19 pandemic.

**Methods:**

This was a cross-sectional study on a sample of Iranian and Afghan women of reproductive age. To collect the data, the 12-item short-form health survey (SF-12) and the Enrich marital satisfaction scale were used to assess the quality of life and marital satisfaction, respectively. In addition, the Global Rating of Change (GRC) was used in order to evaluate the quality of life and marital satisfaction compared to before the COVID-19 pandemic. Data were evaluated descriptively through statistics including sing t-test, and chi-square, Logistic regression was performed to assess the relationship between outcome variables and independent variables.

**Results:**

In all 599 reproductive-age women (300 Iranian, and 299 Afghan) were studied. After adjusting for demographic variables, no significant difference was observed between the two groups for the physical component (P = 0.05) and mental component summary scores of quality of life (*P* = 0.166) as measured by the SF-12. The majority of Iranian women reported that their quality of life was worsened compared to before the pandemic (57.2%), while in the Afghan group, a higher percentage declared that it was unchanged (58.9%). The mental component of quality of life had no significant relationship with any of the independent variables including nationality. In contrast, the physical component quality of life had a significant relationship with nationality (*P* = 0.01). Iranian women had more marital satisfaction than Afghan women (*P<*0.001) and marital satisfaction had a significant relationship with nationality (*P<*0.001). Most women in both groups (70% of Iranian and 60% of Afghan women) declared that their marital satisfaction unchanged compared to before the COVID-19 pandemic.

**Conclusion:**

The results showed that the quality of life of Iranian and Afghan women of reproductive age was almost the same before and after the pandemic. However, Iranians scored lower on the mental component summary and Afghans reported lower scores on the physical component summary. Marital satisfaction of Afghan women was much lower than that of Iranian women. The findings suggest the need for serious attention by health care authorities. Providing a supportive environment might be considered a primary step towards a better quality of life for these populations.

## Introduction

The corona virus disease (COVID-19) pandemic caused by the severe acute respiratory syndrome coronavirus 2 (SARS-CoV-2). The World Health Organization (WHO) declared a Public Health Emergency of International Concern (PHEIC) on 30 January 2020, and characterized it the outbreak as a pandemic on 11 March 2020 [1]. In response to this global health crisis, restrictive and quarantine measures were implemented by international and governmental health organizations to combat the rapid spread of the disease [2].

A wide range of psychological outcomes at individual and social levels have been observed during the outbreak of the disease. At the individual level, people were likely to experience the fear of getting sick or dying, feeling helpless and isolated [3]. Evidence suggest that the prevalence of stress, anxiety, depressive disorder, and insomnia increased during the COVID-19 pandemic [4]. One of the risk factors predicting these complications is women gender [5]. According to recent studies, the prevalence of anxiety, depression, and stress during the covid-19 pandemic were higher in women than in men [6, 7]. In addition, the quarantine and restriction imposed by COVID-19, although they had the impact of reducing contagion, increased the prevalence of depression and anxiety and reduced the quality of life [8].

It is argued that epidemic diseases can cause secondary and heavier effects than the virus itself on the health parameters and quality of life of people [9]. Thus, assessing quality of life and its determinants could provide a general perspective on people’s health status and might play a role in health planning. Currently, quality of life is one of the major concerns of health professionals and is known as an indicator for measuring health status in public health and medical research [10].

As far as marital relationship concerns, quality of life has a mutual effect on marital satisfaction. [11]. Marital satisfaction depends on people's expectations and beliefs, so poor quality of life can hurt marital satisfaction [12]. Low levels of marital satisfaction lead to lower levels of happiness, life satisfaction, self-esteem, and mental health, and generally affects quality of a person life [13, 14]. As such, quality of life and marital satisfaction can be considered as two important factors affecting women's health. Thus, after the spread of mental distress caused by the COVID-19 pandemic, it is necessary to know more about quality of life and its changes in different populations [15]. Since the main investigator interested in studying quality of life in afghan women, it was thought it would be interesting to assess quality of life and marital satisfaction in two neighboring countries that are Iran and Afghanistan. Unfortunately studies on quality of life of Afghan women are scars. There were two studies that addressed quality of life in immigrant afghan women living in Iran. A study in rural Iran, found that the quality of life score and its subscales was comparably low in both Iranian and Afghan pregnant women [16]. Another study showed that after immigration to Iran, the quality of life status of the immigrants has improved significantly [17]. However, there are several studies on quality of life in Iranian women, especially among fertile and infertile women. For instance, a study reported on quality of life among Iranian women of reproductive age and found that higher sexual function and marital satisfaction, higher educational level, and no prior history of depression were predictors of physical and mental component summary scores of health-related quality of life [18].

This study aimed to estimate the global rating changes in quality of life and marital satisfaction (before and after COVID-19) among samples of reproductive age women who were attending to referral centers (teaching hospitals) in Iran and Afghanistan.

## Methods

### Design

This was a descriptive study comparing marital satisfaction and health-related quality of life in women of reproductive age in Iran and Afghanistan during the COVID-19 pandemic in 2021. The study was approved by the Ethics Committee of Tarbiat Modares University (IR.MODARES.REC.1400.093).

### Participants and sampling

Women of reproductive age attending to a teaching hospital affiliated to Tehran University of Medical Sciences in Tehran and a teaching hospital affiliated to Balkh University in Kabul. Women were selected from obstetrics and gynecology clinics. They were included in the study if they were aged between 18 to 45 years, not having prior health threatening diseases, and wishing to participate and be interviewed. The questionnaires used in the study were completed through interviews. Since we thought it might be difficult for women to complete the questionnaires specially for women in Afghanistan and to prevent missing data, after they agreed to participate in the study they were invited to a calm places in designated hospital and interviewed. The interviews were carried out by two trained females and both received online instruction by the main investigator (MKH) how to interview and collect the data.

### Sample size calculation

Considering an overall 15% difference in the global rating change between Iranian and Afghan women (P1 = 50% and P2 = 35% respectively), the minimum sample size was estimated using the following formula:

$$n={(Z_{\alpha/2}+Z_\beta)}^2\ast(p_1(1-p_1)+p_2(1-p_2))/{(p_1-p_2)}^2$$

The type one error and power were considered as 0.05 and 90%, respectively. Therefore, the minimum sample size of 228 women per each group was calculated. However, allowing for possible drop outs and missing data, 288 women per each group was thought. Hopefully in practice a larger sample size was achieved.

### Measures

To collect the data, the following questionnaires were used:

1. **Demographic questionnaire:** This included information on nationality, age, education, job status, estimated family income, number of children, number of abortions, age of marriage, duration of the marriage, and history of infertility.
2. **Health related quality of life:** It was measured by the short form-12 health survey (SF-12v2). The SF-12 questionnaire is a shorter version of the SF-36 health survey [19, 20]. The instrument contains eight subscales as the original 36-item questionnaire: physical functioning (PF, 2 items), role limitations due to physical problems (RP, 2 items), bodily pain (BP, 1 item), general health perceptions (GH, 1 item), vitality (VT, 1 item), social functioning (SF, 1 item), role limitations due to emotional problems (RE, 2 items) and mental health (MH, 2 items) which are summarized in two mental component summary (MCS) and physical component summary(PCS). The score on each subscale and summary scores range from 0 to 100 where the higher scores indicate better conditions. We used the Persian version of the questionnaire. The psychometric properties of the Persian version are well documented [21, 22].
3. **Marital satisfaction:** It was measured by the Enrich Marital Satisfaction Scale. This scale contains 10 questions that are scored on a Likert scale ranging 1 to from 5 (strongly disagree to completely agree). The total score of this questionnaire varies from 10 to 50. Higher scores indicate higher marital satisfaction. The validity and reliability of the Persian version are confirmed [23].
4. **The Global Rating of Change (GRC):** It was used in order to evaluate quality of life and marital satisfaction compared to before the COVID-19 pandemic. The scale asks the person to evaluate her current state of health compared to a specific time in the past. Then it is scored on a numerical scale or numerical analog [24]. Based on the (GRC) at the beginning of the study, the participants also were asked to rate their quality of life and marital satisfaction compared to before the COVID-19 epidemic on a Likert scale (much better = 5, better = 4, no change = 3, worse = 2, and much worse = 1). Although some investigators criticized the use of global rating of change, a recent systematic review with meta-analysis and meta-regression confirmed that evidence from very good-to-excellent quality studies found that global rating of change scores are moderately correlated to an external criterion patient-reported outcome measures [25].

### Statistical analysis

The demographic information of the two groups of Iran and Afghanistan was reported using descriptive statistics (mean, standard deviation, percentage and number). To compare the data, independent t-test, chi-square, and general linear regression were used. The general linear model analysis was used to compare mental component summary, physical component summary, and global rating of change while using nationality and occupation as fixed factors and age, number of children, age at marriage, and duration of marriage as covariates. The reason for such adjustment was due to the fact that the two study groups were significantly differed in these characteristics. In addition, logistic regression analysis was used to investigate the relationship between quality of life and independent variables. Similar analysis also was performed for marital satisfaction. For this purpose, proportional to mean score the mental component summery score, physical component summery score, and marital satisfaction score were categorized into two groups, less than mean, and equal or more than mean (1 and 0, respectively). The logistic regression analyses were performed while controlling for all independent variables listed in Table 1. All the tests were performed using SPSS version 25 software. The significant level was set at *P* < 0.05.

**Table 1:** The demographic characteristic of the participants

	**Iranian (*n* = 300)**		**Afghani (*n* = 299)**		
	**No. (%)**	**Mean (SD)**	**No. (%)**	**Mean (SD)**	***P*-value**
**Age (years)**		34.57(5.72)		27.38(4.77)	<0.001*
**Education (years)**		14.48(3.36)		14.23(2.54)	0.31*
**Occupation**					<0.001**
Housewife	231 (77.0)		68 (22.7)		
Employed	62 (20.7)		159 (53.2)		
Student	7 (2.3)		72 (24.1)		
**Family income**					0.15**
Poor	185(61.7)		204 (68.2)		
Intermediate	100 (33.3)		78 (26.1)		
Good	15 (5.0)		17 (5.7)		
**Marriage age(years)**		23.26(5.2)		20.91(2.8)	<0·001*
**Duration of marriage(years)**		11.08(6.2)		6.5(5.7)	<0·001*
**Number of children**		1.40(0.900)		1.75(1.42)	<0·001*
**Number of abortions**		0.42(0.79)		0.31(0.73)	0.09*
**History of infertility**					0.16**
Yes	33 (11)		23 (7.7)		
No	267 (89)		276 (92.3)		

*Independent t test, **Chi-squared test, No.: Number, SD: Standard Deviation

## Results

### Participants

The characteristics of the study samples are presented in Table 1. Iranian and Afghan groups differed significantly in the following demographic characteristics: age, occupation, age, duration of marriage, and the number of children.

### Quality of life and global change in quality of life

The mental component summary (MCS) and the physical component summary (PCS) of the SF-12 were used to evaluate the quality of life. After adjusting for the effect of demographic variables including nationality and occupation as fixed factors and age, number of children, age at marriage, and duration of marriage as covariates, there was no significant difference neither in the mental component summary (*P* = 0.16) nor in the physical component summary scores (*p* = 0.05).

To evaluate the quality of life compared to before the COVID-19 pandemic, the global rating of changes (GRC) was used. There was a significant difference between the GRC scores in two groups indicating higher score in Afghan women compared to Iranian women (2.75 ± 0.05 and 2.48 ± 0.06, respectively). The detailed results are presented in Table 2.

**Table 2:** Comparison of quality of life and GRC in quality of life compared to the past (before pandemic) in two groups

	**Iranian (*n* = 300)**		**Afghani (*n* = 299)**		
	**Mean (SD)**	**No. (%)**	**Mean (SD)**	**No. (%)**	***P***-**value**
**MCS**	43.42 (0.90)		45.24 (0.77)		0.166*
**PCS**	48.56 (0.62)		46.95 (0.52)		0.05*
**GRC**	2.48 (0.06)		2.75 (0.05)		0.003*
GRC**					<0.001
Worse		172 (57.3)		72 (24)	
Unchanged		111 (37.0)		176 (58.9)	
Better		17 (5.7)		51 (17.1)	

*MCS* mental component summery, *PCS *physical component summery, *GRC *Global Rating of Change in quality of life compared to the past (before pandemic)

^*^Obtained from general linear model analysis to compare MCS, PCS and GRC while using nationality and occupation as fixed factor and age, number of children, age at marriage, and duration of marriage as covariates

^**^Extra analysis of global rating scale as categorical data (Chi-squared test: X^2^ = 72.70, df = 2)

### Relationship between MCS and PCS and independent variables

Logistic regression was used to examine the relationship between two mental and physical components of quality of life and independent variables. For this purpose, MCS and PCS of quality of life were considered as dependent variables and demographic variables and fertility as independent variables. There was no significant relationship between MCS and variables studied. The results are presented in Table 3. However, there was a significant and positive relationship between lower PCS score and nationality (OR for being Afghan: 1.94, 95%CI: 1.43–3.30, *P* < 0.01). The results are shown in Table 4.

**Table 3:** Odds ratio for poor mental component summary adjusted for nationality and independent variables

	**OR (95%CI)**	*P*-value*
**Nationality**		
Iranian	1.00 (ref.)	-
Afghani	0.70 (0.41–1.18)	0.18
**Age** (years)	1.01 (0.94–1.09)	0.63
**Education** (years)	1.04 (0.98–1.12)	0.16
**Occupation**	-	
Employed	1.00 (ref.)	-
Housewife	1.06 (0.67–1.67)	0.78
Student	1.20 (0.65–2.21)	0.54
**Family income**	-	
Good	1.00 (ref.)	-
Intermediate	2.19 (0.99–4.83)	0.05
Poor	1.2(0.53–2.70)	0.65
**Age at marriage** (years)	1.03(0.96–1.1)	0.33
**Duration of marriage** (years)	1.03(0.95–1.10)	0.43
**Number of children**	0.98(0.81–1.19)	0.87
**Number of abortions**	1.04(0.83–1.3)	0.69
**History of infertility**		
No	1.00(ref.)	-
Yes	1.32(0.73–2.39)	0.35

^*^Obtained from multivariable logistic regression analysis

**Table 4:** Odds ratio for poor physical component summary adjusted for nationality and independent variables

	**OR(95%CI)**	*P*-value*
**Nationality**		-
Iranian	1.00(ref.)	-
Afghani	1.94 (1.14–3.30)	0.01
**Age**(years)	1.06(0.98–1.16)	0.13
**Education**(years)	0.99(0.93–1.06)	0.90
**Occupation**	-	-
Employed	1.00(ref.)	-
Housewife	1.14(0.73–1.79)	0.55
Student	1.12(0.62–2.04)	0.69
**Family income**	-	-
Good	1.00(ref.)	-
Intermediate	1.37(0.63–2.97)	0.41
Poor	1.14(0.51–2.52)	0.73
**Age at marriage** (years)	1.04 (0.69–1.13)	0.31
**Duration of marriage** (years)	0.96(0.88–1.04)	0.40
**Number of children**	0.96(0.79–1.16)	0.69
**Number of abortions**	0.92 (0.73–1.15)	0.48
**History of infertility**		-
No	1.00(ref.)	-
Yes	1.01(0.56–1.82)	0.96

^*^Obtained from multivariable logistic regression analysis

### Marital satisfaction and global change in marital satisfaction

The mean marital satisfaction score was (37.17 ± 0.44) in Iranian women while it was 34.11 ± 0. 37 among Afghans (Table 5). However, after adjusting for independent variables, the GRC of marital satisfaction in two groups had a significant difference, and only 19% of Iranian women and 26% of Afghan women reported that their marital satisfaction was worsened, 70% of Iranian women and 60.2% of women Afghans stated unchanged marital satisfaction and 10% of Iranians and 13% of Afghans women reported better condition (Table 5).

**Table 5:** Comparison of marital satisfaction and GRC in marital satisfaction compared to the past (before pandemic) in two groups

	**Iranian (*n* = 300)**		**Afghani (*n* = 299)**		***P*-value**
	**Mean (SD)**	**No. (%)**	**Mean (SD)**	**No. (%)**	
**Enrich marital satisfaction score**	37.17 ± 0.44		34.11 ± 0. 37		< 0.001*
**GRC**	2.99 (0.05)		2.81 (0.04)		0.02 *
**GRC**					0.02**
Worse		57 (19.0)		79 (26.4)	
Unchanged		212 (70.7)		180 (60.2)	
Better		31 (10.3)		40 (13.4)	

*GRC* Global Rating of Change in marital satisfaction compared to the past (before pandemic)

^*^Obtained from general linear model analysis considering nationality and occupation as fix factor and age, number of children, age at marriage, and duration of marriage as covariates

^**^Chi-squared test (X^2^ = 7.31, df = 2)

### Relationship between marital satisfaction and independent variables

There was a significant and positive relationship between the lower level of marital satisfaction and nationality (OR for being Afghan: 3.25, 95% CI: 1.89–5.59, *P* < 0.001). The results are shown in Table 6.

**Table 6:** Relationship between marital satisfaction and independent variables

	**OR(95%CI)**	*P*-value*
**Nationality**		-
Iranian	1.00(ref.)	-
Afghani	3.25(1.89–5.59)	0.001
**Age**(years)	0.99(0.89–1.09)	0.84
**Education**(years)	1.01(0.94–1.08)	0.76
**Occupation**	-	-
Employed	1.00(ref.)	-
Housewife	0.92(0.58–1.45)	0.72
Student	1.48(0.81–2.70)	0.2
**Family income**	-	-
Good	1.00(ref.)	-
Mild	1.26(0.58–2.72)	0.55
Poor	0.89(0.40–1.96)	0.77
**Age at marriage** (years)	1.04 (0.94–1.16)	0.35
**Duration of marriage** (years)	1.06(0.95–1.17)	0.25
**Number of children**	1.12(0.92–1.37)	0.22
**Number of abortions**	1.14 (0.91–1.44)	0.23
**History of infertility**		-
No	1.00(ref.)	-
Yes	0.79(0.43–1.45)	0.45

^*^Obtained from multivariable logistic regression analysis

## Discussion

The findings from the current study indicated that there were no significant differences in mental and physical component summary scores between Iranian and Afghan women although the significance for difference in physical component summary between the two groups was at the threshold level (*P* = 0.05).

A significant number of Iranian women reported that their quality of life was worsened compared to before the pandemic, while in the Afghan group, a large number declared that it was unchanged. This difference in the two groups can probably be explained by the different perceptions of quality of life among women in two countries. In addition, perhaps implementing more restrictions and preventive measures in Iran compared to Afghanistan reflected on such a difference in evaluation of quality of life by the two study samples. Evidence suggest that tougher restrictions due to COVID-19 had stronger influences on people’s quality of life. For instance, a study from Italy aimed at evaluating the effect of social distancing measures due to the COVID-19 pandemic on sexual performance and quality of life of non-infected women of reproductive age in Rome showed a decrease in sexual performance and quality of life of women of reproductive age during social restrictions. According to the results of this study, social restrictions and uncertainty about the future affected people's quality of life and sexual performance [26]. It seems that the above mentioned findings are very similar to what we found in Iran. However, the observed difference for Afghan women can be due to the difference in the implementation of preventive measures where probably fewer social restrictions were applied.

The mental component summary score showed no significant relationship with any of the independent variables, while physical component summary score had a significant relationship with nationality. The likelihood of lower PCS score was 1.94 times higher for being Afghan. Along with cultural and social factors, poor financial status might be a reason why Afghan women scored lower on physical component summary. A study suggested that when one experiencing problems in daily life and might have different social roles, especially for women, health may become a secondary issue [27]. A study assessing factors affecting the quality of life of women in Isfahan, Hachigitian found that social capital has an even greater effect on the quality of life of women than income and job [28]. Considering the limited role and social activity of women in Afghanistan, these factors can help to explain such a difference.

Iranian women had more marital satisfaction than Afghan women and marital satisfaction had a significant relationship with nationality, and for Afghani women were more likely to report lower marital satisfaction (OR = 3.25) compared to Iranian women. There are several factor that might affect marital satisfaction including religious factors, sexual practices, communicative and interactive factors, and mental health. Also, the influence of some socio-demographic factors such as occupation, duration of marriage, age, number of children, economic factors and income are emphasized [29]. Comparison of marital satisfaction in two countries has not been investigated, but due to cultural differences, it is thought that the factors affecting marital satisfaction in Afghanistan are different from Iran. Of course, the social and demographic differences of the two countries, such as economic, security and job factors, should not be ignored. The level of marital satisfaction varies based on the power structure in the family, the family socio-economic status, and women's education. Marital satisfaction is higher in families with a matriarchal structure than in families with a collective and patriarchal structure. They have more dignity and respect in the society, which can also affect the marital satisfaction of a person [30]. It is possible to consider the possible role of these factors in the better marital satisfaction of Iranian women or the lower marital satisfaction of Afghan women.

Iranian and Afghan women evaluated their marital satisfaction differently compared to before the pandemic. Only 19% of Iranian women and 26% of Afghan women reported that their marital satisfaction was worsened than before COVID-19, and most of them (70% of Iranians and 60.2% of Afghans) reported their marital satisfaction unchanged, and 10 and 13% of Iranians and Afghans also reported better marital satisfaction.

Finally, one should note that the current study has some limitations. Firstly, the descriptive nature of the study and the sampling method does not allow generalization. Secondly as we could not reach women in quarantine, the results should be intercepted with caution. Thirdly since we used global rating of change, one should take into account that such a measure could not fully reflect the changes occurred in quality of life and marital satisfaction among the study samples. Perhaps a qualitative study could help to accurately understand how the COVID-19 affected quality of life and marital satisfaction among these populations.

## Conclusion

The results showed that quality of life of Iranian and Afghan women of reproductive age were almost the same before and after the pandemic. However, Iranians scored lower on mental component summary and Afghans reported lower scores on physical component summary. Marital satisfaction of Afghan women was much lower than that of Iranian women. Indeed, the findings reflect the conditions that could be challenging and need serious attention by health care authorities. Providing supportive environment might be considered as a primary step towards a better quality of life and marital life for these populations.

## Data Availability

The data are available from corresponding authors on reasonable request.

## Abbreviations

MCS: Mental Component Summery
PCS: Physical Component Summery
GRC: Global Rating of Change
